# The Costs of Operative Complications for Ankle Fractures: A Case Control Study

**DOI:** 10.1155/2014/709241

**Published:** 2014-11-27

**Authors:** Frank R. Avilucea, Sarah E. Greenberg, W. Jeffrey Grantham, Vasanth Sathiyakumar, Rachel V. Thakore, Samuel K. Nwosu, Kristin R. Archer, William T. Obremskey, Hassan R. Mir, Manish K. Sethi

**Affiliations:** The Vanderbilt Orthopaedic Institute Center for Health Policy, 1215 21st Avenue S., MCE, South Tower, Suite 4200, Vanderbilt University, Nashville, TN 37232, USA

## Abstract

As our healthcare system moves towards bundling payments, it is vital to understand the potential financial implications associated with treatment of surgical complications. Considering that surgical treatment of ankle fractures is common, there remains minimal data relating costs to postsurgical intervention. We aimed to identify costs associated with ankle fracture complications through case-control analysis. Using retrospective analysis at a level I trauma center, 28 patients with isolated ankle fractures who developed complications (cases) were matched with 28 isolated ankle fracture patients without complications (controls) based on ASA score, age, surgery type, and fracture type. Patient charts were reviewed for demographics and complications leading to readmission/reoperation and costs were obtained from the financial department. Wilcoxon tests measured differences in the costs between the cases and controls. 28 out of 439 patients (6.4%) developed complications. Length of stay and median costs were significantly higher for cases than controls. Specifically, differences in total costs existed for infection and hardware-related pain. This is the first study to highlight the considerable costs associated with the treatment of complications due to isolated ankle fractures. Physicians must therefore emphasize methods to control surgical and nonsurgical factors that may impact postoperative complications, especially under a global payment system.

## 1. Introduction

Healthcare costs in the United States were approximately $2.8 trillion in 2012, representing 17.9% of the national gross domestic product [1]. In an effort to curb the rising costs of care, our healthcare system is moving towards a global payment model in which readmissions for complications may not be reimbursed. Given that ankle fractures, occurring at a rate of 187 per 100,000 people each year, are one of the most common injuries treated by orthopaedic surgeons, it is important to understand the costs of treating the surgical complications that can arise from these injuries and the resultant financial implications that may occur under a bundled or global payment system [2].

Unfortunately, even with the high frequency of these injuries, there is minimal literature regarding the financial costs of ankle fracture complications. For example, in an economic analysis by Belatti and Phisitkul, foot and ankle diseases in the Medicare population were estimated to cost $11 billion in 2011, with treatment secondary to fractures contributing to 31% of these costs [3]. However, these authors did not differentiate ankle fractures from other fractures of the foot and did not analyze the costs secondary to complications. Manoukian et al. investigated ankle fracture costs based on the timing of operation in relation to when patients are admitted to the hospital, but their analysis did not focus on complications or specific areas of costs during the hospitalization [4]. Additionally, Murray and colleagues investigated index hospitalization and readmission costs for ankle fracture patients based on type of surgery; however, the authors did not identify reasons for readmission based on type of complication [5].

Despite studies focusing on various aspects of costs related to ankle fractures, to date, no study has investigated the costs related to the types of complications that these patients with ankle fractures may develop. In a bundled-payment system under which these complications may not be reimbursed, it is vital for the practicing orthopaedic surgeon to understand the financial implications associated with post-surgical complications.

To evaluate the costs associated with ankle fracture complications, we designed a case control analysis where we matched patients who developed complications after ankle fracture surgery (“cases”) to those who did not develop complications (controls). The analysis of these cohorts enables us to determine the costs associated with each type of complication. Furthermore, by categorizing the costs associated with different hospital services, we found which factors have the greatest impact on overall expenditures. Therefore, understanding these variations in costs may help drive policymaking to better prepare orthopaedic surgeons for a bundled-payment system.

## 2. Methods

After institutional review board approval, all patients treated at a level I trauma center from January 1, 2000, to January 1, 2010, for ankle fractures were identified through a CPT code search of the university's orthopaedic database. Patient charts were reviewed to include only those who had isolated ankle fractures without any concomitant injuries, including additional fractures, organ injuries, or neurovascular injuries. The charts of these remaining patients were reviewed for basic demographic information including age and gender, as well as type of fracture, fracture location, length of stay for index hospitalization and readmissions, American Society of Anesthesiologist (ASA) score, and complications. Complications included those that resulted in reoperation or readmission and included nonunion, infection, hardware pain, hardware failure, and malunion.

Patients with complications (cases) were matched to those without complications (controls) following principles in orthopaedic literature, which are based on correlating specific factors that may be associated with the outcome of interest. In our study, we matched by ASA score (within one score), age (within 10 years), type of surgery (based on CPT code), and type of fracture (open versus closed) as several studies found correlations between these variables and development of complications [6–8]. Costs for cases and controls were obtained from the university's financial database and included professional and technical charges. Professional charges were subcategorized into costs related to surgery, radiology, and evaluation and management of patients, whereas technical charges included diagnostic, room and board, surgical implant/materials, and pharmacy charges. Wilcoxon ranked-sum tests were performed to compare the differences in costs and length of stay between the case patients and control patients.

## 3. Results

Through a CPT code search, 439 patients presented with isolated ankle fractures, of which 28 developed a post-surgical complication. Basic demographic data, including age, gender, fracture type, and ASA score, for the 28 cases and their corresponding 28 controls is provided in Table 1. The average age of cases was 44 ± 15 years compared to 45 ± 14 years for the control group. Both groups had 15 closed (53.6%) and 13 open (46.4%) fractures, and the average ASA score for cases was 2.14 ± 0.59 compared to 2.21 ± 0.57 for controls. Of the 28 patients with complications, 7 (25.0%) had nonunion, 8 (28.6%) had infection, 10 (35.7%) had hardware pain, 3 (10.7%) had hardware failure, and 1 (3.6%) had malunion.

Table 2 provides the median charges for cases and controls without controlling for type of complication. There were significant differences in median charges between the groups with respect to both professional charges (difference of $8,185) and technical charges (difference of $32,532). Among the professional charges, the median surgical charges specifically differed between the case and control groups ($13,857 and $6,931, resp.). The technical charges associated with implant/materials and pharmacy charges were also significantly higher in the cases when compared to the controls (Table 2). Furthermore, the overall length of stay was greater for the cases (5.5 days) compared to controls (3.0 days) (*P* = 0.028).

**Figure 1:**
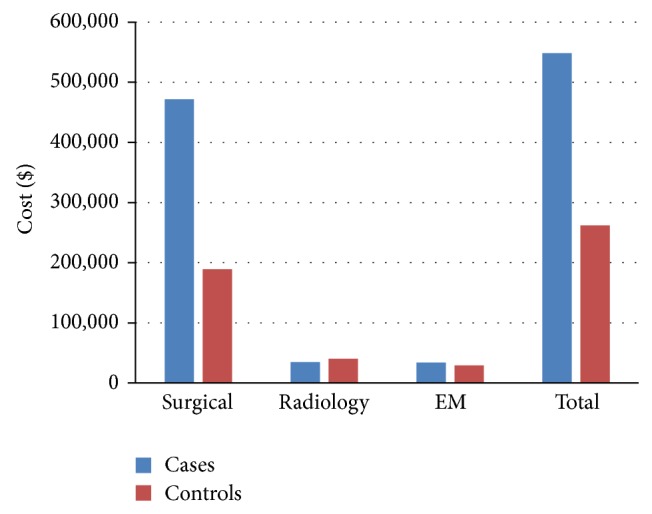
Aggregate professional charges for cases and controls are broken down into surgical, radiology, EM (evaluation and management), and total charges. Median values are reported.

**Figure 2:**
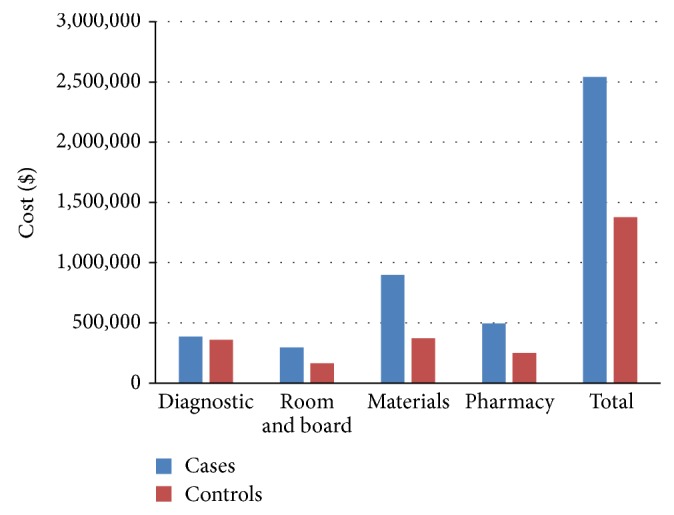
Aggregate technical charges for cases and controls are broken down into diagnostic, room at board (R&B), surgical materials and implants (materials), pharmacy, and total charges. Median values are repotted.

Figures 1 and 2 detail the aggregate professional and technical charges, respectively, for the 28 patients within each group. The cases had higher aggregate costs in each category except for radiology charges. In sum, the 28 cases had an aggregate professional and technical charge of $3,090,331 compared to $1,637,589 for the 28 controls.

Tables 3, 4, 5, and 6 detail costs for matched cases and controls based on type of complication. For nonunions, total professional charges and the subcategory of surgical charges had significantly higher costs associated with the case group compared to the control group ($17,863 versus $8,358, and $16,397 versus $6,888, resp.). For infections, both professional and technical charges were significantly higher in the patients who presented with this complication, resulting in significantly higher total charges, $128,122 for the cases compared to $49,983 for the controls. These significant differences in charges between the groups were driven by greater surgical, room and board, and pharmacy costs for the patients with infections. The length of stay was also 16.0 days for the case group that presented with infection, which was significantly longer than the length of stay calculated for the control group (3.0 days) (*P* ≤ 0.001). Similarly for hardware-related pain, the cases presented with both higher professional and technical charges; however, these costs were mainly due surgical and pharmacy charges, respectively. There were no significant differences in all categories of charges between cases and controls for hardware failure, although only 3 patients had this type of complication. Finally, only 1 patient had a nonunion out of our series of 28 patients; therefore a Wilcoxon ranked test was not run. However, this 1 patient did have greater costs in all categories except radiology ($260 versus $2,045) and diagnostics ($13,160 versus $14,478) compared to a similarly matched patient.

**Figure 3:**
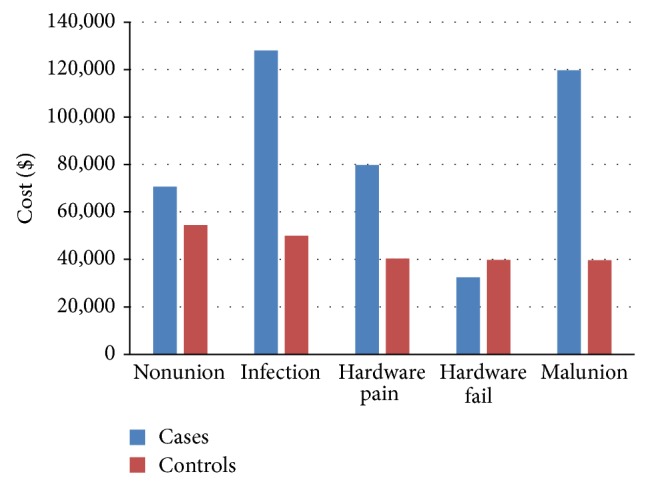
The total charges for each complication are compared to controls. ^*^Malunion: only one patient had a malunion.

Figure 3 provides a comparison of median costs associated with each type of complication. Infection resulted in the greatest expense for patients, whereas hardware failure resulted in the lowest charges.

## 4. Discussion

Our study is the first to report on the specific costs associated with complications related to surgical treatment of patient with an isolated ankle fracture. Based on the results of our study, patients with ankle fractures who develop complications incur significantly greater professional ($16,500 versus $8,300), technical ($69,200 versus $36,300), and total charges ($85,000 versus $60,500) than patients who do not develop complications. Among the specific types of complications, patients who developed infections or had hardware-related pain experienced significantly higher costs compared to their corresponding control patients. Specific subcategories of professional and technical charges, like surgical charges, were higher or lower depending on the type of complication, thereby highlighting areas that may cause the greatest financial risk under a bundled-payment system.

To our knowledge, no other study has compared the costs of all common ankle fracture complications. However, a few studies have investigated costs with certain aspects of ankle fractures and therefore serve as a basis for comparison. In Sanders et al.'s study of 11 open grade IIIB ankle fractures, average inpatient charges were just over $62,000 per patient [9]. This patient cohort consisted of patients with and without complications including soft tissue infection and osteomyelitis. Similarly, in our study we found a median inpatient charge of $61,000 when combining the cases and controls together. Marsh and colleagues investigated 7 patients with supramalleolar nonunions after tibial plafond fractures and found an average patient cost of $66,491 per patient to treat these nonunions [10]. In our analysis, we found a similar result with a slightly higher median value of $70,644 in nonunion patients. Given the similarity of our results to isolated results in the literature, we have some external validation of our data thereby helping the generalizability of our results.

Given that the annual rate of ankle fractures is 187 per 100,000 Americans, then, based on the current US population of approximately 318 million people, nearly 594,650 Americans sustain ankle fractures each year [1, 11]. Based on our study, of those with ankle fractures, about 6.4%, or approximately 38,000 individuals, will develop complications. In fact, this complication rate is conservative compared to other studies that determined complication rates for ankle fractures to be upwards of 30% of patients [11, 12]. Based on the rate of $3 million in aggregate charges per 28 patients with complications in our study, 38,000 complications would cost the US healthcare system $4.1 billion in treatment each year. Complications related to surgical treatment of ankle fractures alone would, therefore, incur a small, yet, significant portion of healthcare costs.

The results of our study identify specific complications that are more likely to increase costs for postsurgical ankle fracture patients. Orthopaedic surgeons should be aware of the high costs associated with treatment of complications including infection. Such a finding emphasizes the importance of preoperative antibiotics and thorough debridement in open fractures, minimizing soft-tissue stripping intraoperatively, and employing 24 hours of postoperative antibiotics following surgical intervention [13, 14].

Although not specifically assessed in this study, the mode of fixation utilized is known to have variable costs. Murray et al. reported that external fixation of ankle fractures results in greater financial expenditures compared to open reduction internal fixation [5]. Moreover, locking plate technology is more costly than a standard plate. It is the opinion of the authors that every case is unique and the mode of fixation should be geared at effectively attaining and maintaining fracture reduction. In an era of cost-containment, not only is the patient selection important, but also choice of implant should render effective maintenance of fracture reduction while concomitantly limiting potential hardware and soft-tissue complications. The role of optimizing patient's medical comorbidities prior to surgical intervention (e.g., glucose control, selectively discontinuing immunosuppressants, addressing tobacco use, ASA) is essential. Finally, room and board or hospital length costs may be decreased through early operations and identifying patients who are predisposed to longer lengths of stay based on well-established risk factors to facilitate discharge [15–17].

Our study had some limitations. For one, our cost calculations were based only on the type of complication. However, other factors impact ankle surgery costs and may confound the relationship between costs and type of complication. Furthermore, while we matched patients on multiple comorbidities, additional factors such as open fracture grade may have been important to control for given the clear link between open fracture grade and infection risk [18]. It may be difficult to fully generalize our results based on the definitions our institution uses in categorizing costs. As some areas like malunion only had one patient with an isolated injury, it will be important to repeat our analysis with a larger cohort of patients. Additionally, as this study was completed in an academic medical center, the involvement of residents in patient care may bring into question whether this is an independent risk factor for postoperative complication. Although the evaluation of such trainee involvement has predominantly been addressed in the general surgery literature, a recent report of 13,109 total hip arthroplasty cases shows no increased complication rates within the first 30 days following primary [19–24]. Given the numerous reports as well as the aforementioned large-scale study, resident involvement in these procedures should continue to be encouraged and cultivated. Albeit no surgical intervention is without risk of post-surgical complication, patient and surgeon factors should be mitigated to enable a complication-free outcome.

Our study determined that complications due to isolated ankle fractures greatly increase the cost of treatment. Even though complications cannot be completely prevented in surgery, practicing orthopaedic surgeons should remain vigilant in minimizing factors that potentially lead to complications. Overall, by finding avenues to reduce complications, orthopaedic surgeons can also minimize charges under a bundled-payment system.

## Figures and Tables

**Table 1:** Selected demographics for cases and controls.

	Cases (*n* = 28)	Controls (*n* = 28)
Age (years)		
10–20	1 (4%)	2 (7%)
21–30	5 (18%)	2 (7%)
31–40	5 (18%)	6 (21%)
41–50	7 (25%)	7 (25%)
51–60	6 (21%)	9 (32%)
61–70	3 (11%)	1 (4%)
>70	1 (4%)	1 (4%)
Gender		
Male	13 (46%)	13 (46%)
Female	15 (54%)	15 (54%)
Fracture type		
Closed	13 (46%)	13 (46%)
Open	15 (54%)	15 (54%)
ASA score		
1	2 (7%)	2 (7%)
2	18 (64%)	18 (64%)
3	7 (25%)	8 (29%)
4	1 (4%)	0 (0%)

**Table 2:** Case versus control comparison for *overall* costs.

	Overall costs (median, IQR)		*P*
	Cases (*n* = 28)	Controls (*n* = 28)	
Professional charges	$16,454 ($13,497–$26,648)	$8,269 ($6,554–$11,063)	<0.001
Surgical	$13,857 ($11,269–$22,298)	$6,931 ($4,290–$8,187)	<0.001
Radiology	$688 ($330–$2,080)	$1,789 ($245–$2,197)	0.940
Evaluation and management	$892 ($573–$1,101)	$669 ($501–$997)	0.240
Technical charges	$69,162 ($39,849–$112,153)	$36,630 ($31,093–$57,418)	0.002
Diagnostic	$12,169 ($3,425–$20,920)	$12,446 ($3,339–$18,007)	0.730
Room and board	$4,845 ($1,354–$14,538)	$2,193 ($1,612–$4,800)	0.200
Implant/materials	$25,522 ($11,888–$42,816)	$15,244 ($9,551–$25,754)	0.002
Pharmacy	$12,576 ($5,525–$22,132)	$7,495 ($5,078–$14,527)	0.006
Total charges	$84,980 ($53,254–$139,950)	$60,538 ($40,606–$98,484)	0.001

**Table 3:** Case versus control comparison for *nonunion* costs.

	Nonunion costs (median, IQR)		*P*
	Cases (*n* = 7)	Controls (*n* = 7)	
Professional charges	$17,863 ($15,683–$30,348)	$8,358 ($6,679–$14,729)	0.028
Surgical	$16,397 ($14,572–$25,742)	$6,888 ($3,620–$11,481)	0.003
Radiology	$499 ($347–$1,354)	$2,102 ($1,444–$2,276)	0.190
Evaluation and management	$765 ($500–$1,556)	$785 ($540–$1,066)	0.860
Technical charges	$50,508 ($39,405–$141,563)	$46,346 ($38,815–$57,597)	0.360
Room and board	$3,530 ($2,016–$11,560)	$2,328 ($1,031–3,442)	0.390
Implant/materials	$25,073 ($13,862–51,470)	$11,210 ($7,260–$20,102)	0.084
Pharmacy	$5,787 ($5,495–$27,102)	$7,011 ($5,329–$7,495)	0.500
Total charges	$70,644 ($54,110–$170,775)	$54,453 ($49,196–$68,750)	0.240

**Table 4:** Case versus control comparison for *infection* costs.

	Infection costs (median, IQR)		*P*
	Cases (*n* = 8)	Controls (*n* = 8)	
Professional charges	$25,186 ($14,163–$27,900)	$10,698 ($8,020–$11,208)	0.004
Surgical	$19,906 ($10,949–$24,480)	$7,947 ($7,069–$9,912)	0.010
Radiology	$1,566 ($446–$2,080)	$724 ($140–$1,835)	0.360
Evaluation and management	$898 ($603–$1,494)	$716 ($315–$952)	0.220
Technical charges	$107,549 ($80,424–$161,665)	$39,068 ($31,924–$51,639)	0.006
Diagnostic	$18,084 ($11,152–$23,253)	$9,137 ($4,118–$13,194)	0.072
Room and board	$12,544 ($6,952–$19,894)	$2,058 ($2,031–$3,458)	0.041
Implant/materials	$31,059 ($21,701–$45,540)	$17,252 ($14,351–$19,285)	0.093
Pharmacy	$23,554 ($19,325–$31,730)	$8,254 ($5,547–$9,699)	0.004
Total charges	$128,122 ($106,211–$183,697)	$49,983 ($42,249–$60,529)	0.006

**Table 5:** Case versus control comparison for *hardware pain* costs.

	Hardware pain costs (median, IQR)		*P*
	Cases (*n* = 10)	Controls (*n* = 10)	
Professional charges	$13,714 ($12,030–$16,876)	$8,101 ($6,121–$8,960)	0.006
Surgical	$12,216 ($10,888–$12,644)	$4,478 ($3,939–$7,051)	0.003
Radiology	$1,424 ($391–$2,404)	$1,832 ($288–$2,177)	0.510
Evaluation and management	$936 ($677–$1,091)	$614 ($570–$941)	0.470
Technical charges	$61,165 ($44,829–$76,314)	$32,793 ($27,463–$52,029)	0.030
Diagnostic	$14,420 ($2,434–$21,170)	$12,100 ($2,766–$17,206)	0.660
Room and board	$3,502 ($1,379–$14,094)	$2,015 ($1,461–$4,092)	0.890
Implant/materials	$25,111 ($14,822–$31,141)	$10,560 ($9,360–$13,996)	0.019
Pharmacy	$11,295 ($9,200–$12,829)	$5,725 ($4,969–$7,236)	0.030
Total charges	$79,747 ($56,243–93,399)	$40,360 ($35,223–$60,591)	0.037

**Table 6:** Case versus control comparison for *hardware failure* costs.

	Hardware failure costs (median, IQR)		*P*
	Cases (*n* = 3)	Controls (*n* = 3)	
Professional charges	$16,012 ($11,892–$22,453)	$7,231 ($6,120–$13,571)	0.33
Technical charges	$24,106 ($20,259–$72,818)	$32,539 ($24,799–$131,982)	0.57
Surgical	$14,871 ($10,919–$19,726)	$4,957 ($4,927–$6,227)	0.14
Radiology	$272 ($253–302)	$1,614 ($862–$2,861)	0.57
Evaluation and management	$769 ($633–$1,760)	$660 ($330–$4,450)	0.85
Diagnostic	$3,618 ($3,232–$8,159)	$12,125 ($6,746–$29,124)	0.85
Room and board	$651 ($626–$4,758)	$5,250 ($3,602–$24,375)	0.33
Implant/materials	$7,832 ($4,815–$35,174)	$7,514 ($7,186–$8,916)	0.85
Pharmacy	$3,565 ($3,487–$12,679)	$4,623 ($3,944–$28,610)	0.85
Total charges	$32,425 ($31,151–$91,424)	$39,770 ($30,919–$145,553)	0.85
